# Electrical Storm Has Worse Prognosis Compared to Sustained Ventricular Tachycardia after VT Ablation

**DOI:** 10.3390/jcm12072730

**Published:** 2023-04-06

**Authors:** Julian Mueller, Ivaylo Chakarov, Philipp Halbfass, Karin Nentwich, Elena Ene, Artur Berkovitz, Kai Sonne, Sebastian Barth, Christian Waechter, Tobias Schupp, Michael Behnes, Ibrahim Akin, Thomas Deneke

**Affiliations:** 1 Clinic for Interventional Electrophysiology, Heart Centre Bad Neustadt, 97616 Bad Neustadt a. d. Saale, Germany; 2 Department of Cardiology and Angiology, Philipps-University Marburg, 35037 Marburg, Germany; 3 Department of Cardiology, Klinikum Oldenburg, European Medical School Oldenburg-Groningen, Carl von Ossietzky University, 26129 Oldenburg, Germany; 4 First Department of Medicine, University Medical Centre Mannheim (UMM), 68167 Mannheim, Germany

**Keywords:** electrical storm, coronary artery disease, acute heart failure, sudden cardiac death, MACE, mortality, hospitalization

## Abstract

Background: Electrical storm (ES) represents a serious heart rhythm disorder. This study investigates the impact of ES on acute ablation success and long-term outcomes after VT ablation compared to non-ES patients. Methods: In this large single-centre study, patients presenting with ES and undergoing VT ablation from June 2018 to April 2021 were compared to patients undergoing VT ablation due to ventricular tachyarrhythmias but without ES. The primary prognostic outcome was VT recurrence, and secondary endpoints were rehospitalization rates and cardiovascular mortality, all after a median follow-up of 22 months. Results: A total of 311 patients underwent a first VT ablation due to ventricular tachyarrhythmias and were included (63 ± 14 years; 86% male). Of these, 108 presented with ES. In the ES cohort, dilated cardiomyopathy as underlying heart disease was significantly higher (*p* = 0.008). Major complications were equal across both groups (all *p* > 0.05). Ablation of the clinical VT was achieved in 94% of all patients (*p* > 0.05). Noninducibility of any VT was achieved in 91% without ES and in 76% with ES (*p* = 0.001). Patients with ES revealed increased VT recurrence rates during follow-up (65% vs. 40%; log rank *p* = 0.001; HR 1.841, 95% CI 1.289–2.628; *p* = 0.001). Furthermore, ES patients suffered from increased rehospitalization rates (73% vs. 48%; log rank *p* = 0.001; HR 1.948, 95% CI 1.415–2.682; *p* = 0.001) and cardiovascular mortality (18% vs. 9%; log rank *p* = 0.045; HR 1.948, 95% CI 1.004–3.780; *p* = 0.049). After multivariable adjustment, ES was a strong independent predictor of VT recurrence and rehospitalization rates, but not for mortality. In a propensity score-matched cohort, patients with ES still had a higher risk of VT recurrences and rehospitalizations compared to non-ES patients. Conclusions: VT ablation in patients with ES is challenging and these patients reveal the highest risk for recurrent VTs, rehospitalization and cardiovascular mortality. These patients need close follow-ups and optimal guideline-directed therapy.

## 1. Introduction

Ventricular tachyarrhythmias represent severe and life-threatening heart rhythm disorders requiring immediate therapy. Electrical storm (ES) is defined as more than three distinct episodes of sustained ventricular fibrillation (VF) or ventricular tachycardia (VT) requiring therapy within 24 h [1]. The incidence of ES episodes is increasing gradually due to worldwide increasing numbers of patients with implantable cardioverter defibrillator (ICDs). Today, the prevalence in ICD recipients is estimated at 20% [2] with a lower prevalence of 4% in primary preventive and up to 28% in secondary preventive ICD recipients [3,4,5]. While acute mortality is unclear, ES is associated with an increased 12-month mortality of up to 40% [6].

ES might result from a complex interplay between pre-existing pathological conditions creating a vulnerable electrical substrate and acute patient-specific initiating factors. Aberrant Ca^2+^-handling and ionic imbalances have been identified as major contributors to the susceptibly and initiation of malignant ventricular tachyarrhythmias. New onset or worsening of heart failure, changes in antiarrhythmic drug therapies, psychological stress, diarrhoea, hypokalaemia or further comorbidities represent potential triggers for ES, whereas severe systolic dysfunction, chronic kidney disease and VT as an initial arrhythmia are regarded as independent and established predictors for ES [2,4].

Catheter ablation (CA) has been shown to represent an effective treatment option of sustained VTs based on structural cardiomyopathies, reducing VT burden as well as appropriate ICD therapies among these patients [7]. The long-term success rates of CA in ES patients with non-ischemic cardiomyopathy (NICM) seem to equal those among ischemic cardiomyopathy (ICM) ES patients [8]. However, so far, data about the outcomes of CA of ES compared to patients with ventricular tachyarrhythmias but without ES are limited. Therefore, the present study aims to investigate the long-term prognostic impact of CA of ES in consecutive patients compared to patients with ventricular tachyarrhythmias but without ES on VT recurrences, rehospitalization and cardiovascular mortality rates.

## 2. Methods

### 2.1. Study Population

This study included all consecutive patients presenting with drug-refractory ES or at least one sustained VT referred for CA from January 2018 until April 2021 at one institution. ES was defined as ≥3 episodes of ventricular tachyarrhythmias delimited by at least 5 min leading to appropriate ICD therapy during a single 24 h time period or as incessant VT > 12 h [1]. Patients with idiopathic ventricular fibrillation or ventricular fibrillation triggers from Purkinje tissues at the border zone of myocardial infarction were excluded from this analysis. Each patient was counted only once for inclusion during their first interventional CA of VT. Patients with ES were compared to patients with sustained VTs but without ES.

VT was documented using ICD and, in some cases, additionally using 12-lead electrocardiogram (ECG), ECG tele-monitoring or, in case of unstable course or during resuscitation, using external defibrillator monitoring [1]. The definition of ICM was based on the presence of clinical coronary artery disease and/or previous myocardial infarction. Furthermore, patients with MRI images representing cardioembolic myocardial infarction were included in this group. Other structural heart diseases were dilative cardiomyopathy (DCM), hypertrophic (obstructive) cardiomyopathy (HCM), arrhythmogenic right ventricular cardiomyopathy (ARVC), myocarditis and cardiac sarcoidosis. All pathologies were defined according to the respective European guidelines [7,9]. Patients with toxic cardiomyopathy, tachycardia-induced cardiomyopathy and primary valvular abnormalities were excluded.

Values of left ventricular ejection fraction (LVEF) were retrieved from standardized transthoracic echocardiographic examinations usually performed before hospital discharge in survivors to assess realistic LVEF values beyond the acute phase of myocardial ischemia or ES. In a minority of cases and only if available, earlier LVEF values assessed on admission or during intensive care were retrieved from patients who had already died within the acute phase of ES. The documentation period lasted from index VT ablation until October 2021.

All patients gave written informed consent to the ablation procedure and all pre- and post-ablation diagnostics. The study was carried out according to the principles of the Declaration of Helsinki and was approved by the local medical ethics committee of the Heart Centre Bad Neustadt, Germany. All patients gave informed consent for participation in this retrospective analysis.

### 2.2. Electrophysiological Study

All patients underwent VT ablation in the fasting state and under analgosedation using continuous propofol infusion in conjunction with morphine derivates. General anaesthesia was only used when necessary, at the discretion of the operator and only in a minority of cases (<5%). All procedures were performed using a high-density three-dimensional electroanatomic mapping system (CARTO 3, BiosenseWebster, Diamond Bar, CA, USA; Ensite Precision, Abbott, St. Paul, MN, USA; Rhythmia, Boston Scientific, Natick, MA, USA).

According to our standard approach, a high-density voltage map was acquired using a high-density multipolar mapping catheter (Pentaray, BiosenseWebster, Diamond Bar, CA, USA; Advisor HD Grid, Abbott, St. Paul, MN, USA; Intellamap Orion, Boston Scientific, Natick, MA, USA). Areas with bipolar voltage values ≤ 0.5 mV were defined as scar and areas of ≤1.5 mv but >0.5 mV as low-voltage areas, as initially defined by Marchlinski et al. [10]. This definition was applied uniformly to voltage maps acquired using single-tip or multipolar high-density mapping catheters. Local abnormal ventricular potentials, late potentials and fractionated low amplitude potentials were additional criteria for the identification of abnormal pathological ventricular tissue [11].

In some cases, an anterior epicardial approach was adopted using the percutaneous subxiphoid approach described by Sosa et al. [12]. In case of planned epicardial puncture, oral anticoagulation with non-vitamin-K oral antagonists (NOACs) was stopped the day before the procedure, and Vitamin K antagonists (VKAs) were stopped several days before the procedure in order to reach an INR level below 1.5.

Catheters were advanced in the right ventricle (transvenous approach), left ventricle (retrograde access through aortic valve or transseptal approach through mitral valve) or both routes according to the presumed site of VT origin or localization of substrate on cardiac imaging. Programmed ventricular stimulation was performed according to our standard procedure from at least 2 sites using up to 4 extra stimuli with two cycle lengths (CL). In advance, documented 12-lead ECGs of VT morphology were compared to intraprocedural inducible VTs. Induced VTs were counted as “clinical” if they matched the CL and morphology of the recorded 12-lead ECGs or stored ICD electrograms. Inducible VTs were mapped and the critical isthmus was targeted during hemodynamically tolerated VTs. Otherwise, all identified local abnormal activity and late potentials within the scar or low-voltage areas were ablated using 45 Watts and a target ablation index value of 700–1000 in cases with CARTO. In cases with Ensite or Rhythmia, power was titrated to an impedance drop of at least 10% of the baseline value and ablation duration was at least 2 min per lesion. In general, ablation was continued until local electrograms were eliminated. Catheter ablation was performed using an open-irrigated ablation catheter with or without contact force measurement (ThermoCool SmartTouch SF, BiosenseWebster, Diamond Bar, CA, USA; or Tacticath, Abbott, St. Paul, MN, USA; or Intellanav MIFI XP ablation catheter, Natick, MA, USA). The endpoint of catheter ablation was non-inducibility of the clinical VT and it was defined as partial short-term success if other VTs were still inducible. The elimination of any inducible VT was defined as complete short-term success. The ablation of all mapped local abnormal activities and late potentials within a reasonable procedure time was pursued. Endpoint evaluation using programmed ventricular stimulation in the RV and LV was performed in most patients at the end of the ablation process at the discretion of the operator.

### 2.3. Image Integration of Pre-Procedural Cardiac MRI or CTs

According to our standard approach in hemodynamically stable patients, a cardiac computed tomograph (CT) or late gadolinium enhancement-magnetic resonance image (LGE-MRI) was acquired before the procedure if not contraindicated and processed using dedicated software. Processed 3D reconstructions of the ventricles were merged with the 3D electro anatomical mapping system using anatomical landmarks intra-procedurally. Abnormal potentials such as late or fractionated potentials or local abnormal ventricular activities at sites of imaging defined potential conducting channels and were targeted in the first line. Afterwards, all remaining abnormal potentials were also targeted.

Cardiac CT was performed as an arterial phase, end-diastolic acquisition on a wide-detector CT (Revolution CT, GE Healthcare, Chicago, IL, USA) with a spatial resolution of 0.625 mm. Short- and long-axis images with an overlapping slice thickness of 4 mm were calculated from the image data. A scar was assessed if a thinning of the myocardial wall < 4 mm, a band-shaped fatty deposit or calcification could be depicted.

Cardiac MRI was acquired as a late gadolinium 3D whole heart sequence in transverse orientation (slice thickness 2.5 mm, no gap) on a 1.5 tesla scanner (Avanto Fit, Siemens, Munich, Germany). As before, short- and long-axis images with an overlapping slice thickness of 4 mm were calculated. A scar was assessed if a contiguous area with typical signal increase could be defined within the myocardium.

### 2.4. Post-Ablation Management

Patients were monitored until discharge from the hospital and at least 48 h after ablation. Antiarrhythmic drugs were prescribed as needed and at the discretion of the operator. After epicardial ablation, an epicardial drain was inserted in the pericardial space and left in place for at least 12 h or longer in case of protracting exudation.

Post-ablation ICD programming typically included the slowest clinical and/or induced VT zone. In some patients, a (non-)invasive programmed ventricular stimulation (NIPS) before hospital discharge was conducted. Recurrent clinical VT was considered if a VT with a CL equal to or longer than the clinical VT on ICD or having the same QRS morphology on event 12-lead ECG was documented.

### 2.5. Prognostic Study Endpoints

The primary endpoint was recurrence of any sustained ventricular arrhythmia (VA) documented either through ICD interrogations during follow-up in most cases or ECG.

Secondary endpoints comprised first rehospitalization and cardiovascular mortality. First rehospitalization was related to recurrent VT and VF, as well as related to acute heart failure (AHF), acute myocardial infarction (AMI), stroke, left ventricular assist device (LVAD) implantation or heart transplantation.

Cardiovascular mortality was documented using our electronic hospital information system. Furthermore, telephone interviews with patients or family members were performed at the end of the observational period to confirm the absence of primary and secondary endpoints.

### 2.6. Statistical Methods

Quantitative data are presented as mean ± standard error of mean (SEM), median, interquartile range (IQR) and ranges, depending on the distribution of the data, and were compared using Student’s *t* test for normally distributed data or the Mann–Whitney *U* test for nonparametric data. Deviations from a Gaussian distribution were tested using the Kolmogorov–Smirnov test. Spearman’s rank correlation for nonparametric data was used to test univariate correlations. Qualitative data are presented as absolute and relative frequencies and compared using the chi^2^ test or the Fisher’s exact test, as appropriate.

The following analyses were applied stepwise to evaluate the prognostic value of the predefined variables on study endpoints: Kaplan–Meier survival curves were calculated with log-rank testing for statistical significance. Multivariable Cox regression models with VT recurrence, rehospitalization and long-term cardiovascular mortality as the dependent variables were developed using the “forward selection” option, where clinically relevant and univariate statistically significant variables were included and analysed simultaneously.

Propensity score matching was applied using data from the entire patient cohort. We used 1:1 propensity scores for ES versus VT patients to assemble matched and well-balanced subgroups. A one-to-one ratio for propensity score matching was performed applying a non-parsimonious multivariable logistic regression model.

Propensity scores were created according to the presence of the following independent variables: DCM, LVEF, epicardial ablation, full ablation process and CRT device. Uni-variable stratification was performed using the Kaplan–Meier method with comparisons between groups using uni-variable hazard ratios (HR) given together with 95% confidence intervals.

The result of a statistical test was considered significant for *p* < 0.050, and a statistical trend was defined as *p* < 0.100. SAS, release 9.4 (SAS Institute Inc., Cary, NC, USA), was used for statistical analysis.

## 3. Results

### 3.1. Baseline Characteristics

A total of 311 consecutive patients with at least one episode of sustained VT requiring a first VT ablation procedure were included between January 2018 and April 2021 (63 ± 14 years; 86% male). Detailed patient characteristics are presented in Table 1. 108 patients (35%) presented with ES and 203 patients had sustained VTs, requiring ICD therapy or electrical cardioversion in most cases. Both groups were comparable in terms of cardiovascular risk factors as well as comorbidities. The underlying structural heart diseases were comparable except for DCM, which was present significantly more often among patients with ES (31% vs. 17%; *p* = 0.007). ES patients revealed worse LVEF (33 ± 14 vs. 39 ± 15; *p* = 0.001) with concomitant higher rates of already implanted CRT-D systems (25% vs. 12%; *p* = 0.003). Furthermore, ES patients’ medication on admission contained amiodaron (54% vs. 23%; *p* = 0.001) and other antiarrhythmic drugs (AADs) (6% vs. 1%; *p* = 0.021) more often. We found no differences in regard to cardiac biomarkers such as troponins and proBNP upon hospital admission, but potassium levels were significantly decreased among patients with ES.

After propensity score matching, we achieved two well-balanced groups of patients with and without ES with comparable baseline characteristics. Patients with ES had higher rates of amiodarone treatment during hospital admission (50% vs. 29%; *p* = 0.04), whereas non-ES patients had higher rates of liver cirrhosis (0% vs. 4%; *p* = 0.044) and potassium levels (4.50 ± 0.49 mmol/L vs. 4.30 ± 0.57 mmol/L; *p* = 0.014) (Table 1).

### 3.2. Catheter Ablation and Acute Procedural Outcomes

The median time between first VT and the ablation procedure was 25 days, with less time delay for ES patients (18 ± 24 days vs. 28 ± 35 days; *p* = 0.004). For pre-procedural characterization of the ventricular substrate, planning access site (endocardial/epicardial, transseptal/retrograde) and guidance of intraprocedural substrate mapping cardiac CTs and LGE-MRIs were used whenever patients were hemodynamically stable and no contraindications were present. In ES patients, pre-procedural imaging was performed less often, especially cardiac CT (19% vs. 34%; *p* = 0.007).

Epicardial access was obtained in 21% of all patients (29% vs. 16%; *p* = 0.009) and epicardial ablation was performed significantly more often in ES patients (27% vs. 13%; *p* = 0.001). Non-ES patients were more often non-inducible before ablation (28% vs. 16%; *p* = 0.019), whereas ES patients showed higher mean inducible VT numbers (1.9 ± 1.6 vs. 1.5 ± 1.5; *p* = 0.033). Overall procedure and ablation time did not differ among groups; however, longer fluoroscopy times could be documented among ES patients (15.3 ± 10.0 vs. 12.3 ± 8.3; *p* = 0.018). During the ablation procedure, general anaesthesia (8% vs. 1%; *p* = 0.004) and the use of catecholamine (16% vs. 5%; *p* = 0.001) were necessary more often in ES patients.

Acute partial procedural success was high, with non-inducibility of the clinical VT in 95% of all patients (*p* = 0.663). However, in ES patients, full ablation success could be achieved significantly less often (=non-inducibility of any VT) (76% vs. 91%; *p* = 0.001). Accordingly, ES patients were more often discharged from the hospital with amiodarone (41% vs. 19%; *p* = 0.001) (Table 2).

After propensity score matching, procedural characteristics were also comparable between both groups, except patients without ES received preprocedural imaging using MRI more often (33% vs. 17%; *p* = 0.017) (Table 2).

### 3.3. Predictors of Acute Ablation Success

**Figure 1:**
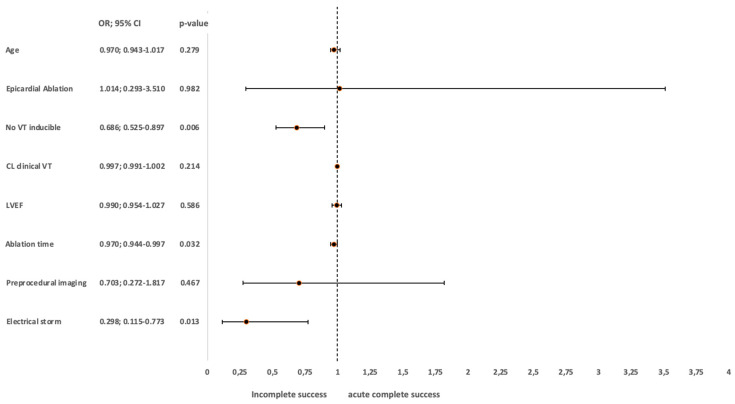
Multivariate logistic regression for the predictors of complete short-term success after catheter ablation of VT in ES and non-ES patients. CL, cycle length; LVEF, left ventricular ejection fraction; No. VT, number of VTs induced during the ablation; and VT CL, VT cycle length in milliseconds.

In the whole cohort, ES was identified as the strongest negative independent predictor of acute ablation success. With the presence of ES, the complete short-term success of VT ablation decreased with a factor of 0.3 (OR 0.298, 95% CI 0.115–0.773; *p* = 0.013). Increased ablation duration also decreased the acute ablation success (OR 0.970, 95% CI 0.944–0.997; *p* = 0.032). For each VT inducible during the procedure, the likelihood for complete short-term success decreased by a factor of 0.7 (OR 0.686, 95% CI 0.525–0.897; *p* = 0.006). Age, epicardial ablation, cycle length of the clinical VT, LVEF and preprocedural imaging were not independent predictors of successful ablation in our model (Figure 1).

### 3.4. Adverse Events during Ablation Procedure

In total, 26 major complications (9%) were recorded, with no difference between both groups. Expectedly, ablation-induced post-interventional third-degree AV block occurred in nine patients, with no difference between both groups. All patients had documented prior AV conduction delay/disturbances and were protected by dual or biventricular pacing devices. Furthermore, in a minority of cases, vascular access-related complications (three patients), pneumonia (four patients), cardiogenic shock (five patients), pneumothorax (three patients) and stroke (two patients) occurred, all comparably distributed between groups. No pericardial tamponade was observed.

In total, three patients died during index hospitalization as procedure-related mortality (two with ES, one without ES). Two patients died due to protracted cardiogenic shock and one patient due to sepsis as part of aspiration pneumonia, 1, 3 and 12 days after the procedure (Table 3).

### 3.5. Long-Term Success Rate and Cardiovascular Mortality

The median follow-up time was 22 months. VT recurrences after the follow-up time were significantly worse in ES patients (40% vs. 65%; log rank *p* = 0.001). ES patients showed a 1.8 times increased risk for VT recurrence in univariate analysis (HR 1.841, 95% CI 1.289–2.628; *p* = 0.001). It should be emphasized that both groups show steep slopes at the beginning of follow-up, representing the high recurrence rates early after VT ablation. Early re-ablation during index hospital stay was performed in 10 patients (5%) (3% vs. 2%; *p* = 1.000). In total, 14% of all ES patients revealed ES recurrences during follow-up, but without any impact on long-term mortality (13% for patients with ES-recurrence, 16% without ES-recurrence; *p* = 0.289). After multivariable adjustment for relevant cofounders, the hazard ratio for VT recurrence was 1.6 (HR 1.621, 95% CI 1.112–2.362; *p* = 0.012). Other independent predictors of VT recurrence were partially successful ablation (any VT inducible at the end of the procedure) (HR 1.590, 95% CI 1.016–2.488; *p* = 0.042) and DCM as underlying heart disease (HR 1.808, 95% CI 1.207–2.709; *p* = 0.004).

Accordingly, ES patients also had increasing rehospitalization rates (73% vs. 48%; log rank *p* = 0.001; HR 1.948, 95% CI 1.415–2.682; *p* = 0.001), mainly driven by rehospitalization due to VT recurrence (54% vs. 32%; *p* = 0.001). Apart from ES (HR 1.582, 95% CI 1.127–2.219; *p* = 0.008), decreased LVEF (HR 1.032, 95% CI 1.010–1.045; *p* = 0.009) was also a multivariable predictor of rehospitalization. Preprocedural imaging was associated with favourable rehospitalization rates (HR 0.630, 95% CI 0.453–0.876; *p* = 0.006).

**Figure 2:**
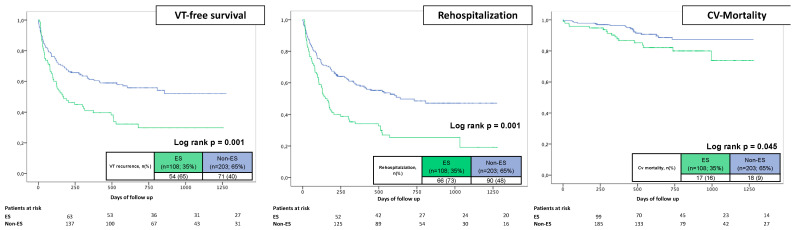
Prognostic impact of ES on VT recurrences (**left** panel), rehospitalization rates (**middle** panel) and cardiovascular mortality (**right** panel).

**Figure 3:**
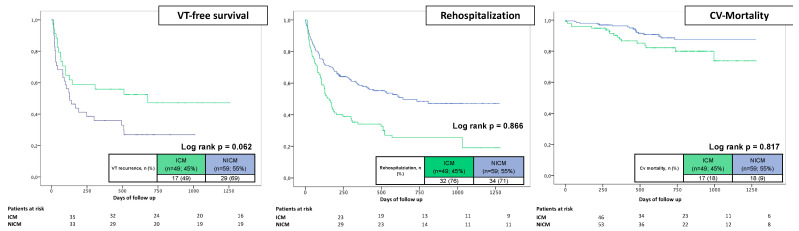
Prognostic impact of ICM and NICM on VT recurrences (**left** panel), rehospitalization rates (**middle** panel) and cardiovascular mortality (**right** panel) in ES patients.

During the follow-up period, cardiovascular death occurred in 11% of all patients. ES patients were significantly more often affected (16% vs. 9%; log rank *p* = 0.045; HR 1.948, 95% CI 1.004–3.780; *p* = 0.049). This finding did not persist in multivariate analysis, where age (HR 1.101, 95% CI 1.054–1.149; *p* = 0.001) and decreased LVEF (HR 1.102, 95% CI 1.023–1.278; *p* = 0.004) were relevant predictors of cardiovascular death. Preprocedural imaging decreased the probability of cardiovascular death in our model (HR 0.361, 95% CI 0.155–0.844; *p* = 0.019) (Table 4 and Table 5; Figure 1 and Figure 2). Notably, in our cohort, full ablation success in ES patients did not significantly influence mortality rates (13% for full ablation success vs. 24% for non-full ablation success; *p* = 0.222) (Figure 3).

### 3.6. Propensity Score-Matched Cohort

**Figure 4:**
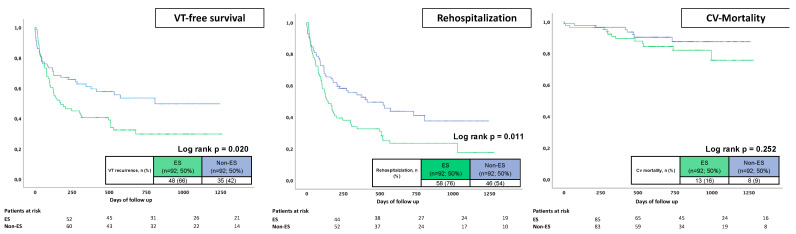
Prognostic impact of ES on VT recurrences (**left** panel), rehospitalization rates (**middle** panel) and cardiovascular mortality (**right** panel) in a propensity score-matched cohort.

After propensity score matching and with comparable baseline and procedural characteristics, patients with ES still showed increasing rates of VT recurrence during follow-up (66% vs. 42%; log-rank *p* = 0.020) (Figure 3). Furthermore, the rehospitalization rates of ES patients were also increased (76% vs. 54%; log-rank *p* = 0.011), mainly driven by VT recurrences and acute heart failure. Cardiovascular mortality rates did not reveal statistically significant differences between both groups after propensity matching (16% vs. 9%; log-rank *p* = 0.252) (Figure 4).

Prognostic value of (non-)invasive programmed ventricular stimulation (NIPS) before discharge.

In 93 patients (30% with ES, 32% without ES), (non-)invasive programmed ventricular stimulation (NIPS) before discharge (mean 3 days after VT ablation; IQR 2–4 days) was performed, in which VT was significantly more often inducible among ES patients (40% vs. 19%; *p* = 0.028). VT inducibility at the end of the VT ablation procedure and in NIPS before discharge strongly correlated for the overall cohort (r = −0.255; *p* = 0.014), but only remained significant among non-ES patients (r = −0.374; *p* = 0.004) (for ES patients, r = −0.099; *p* = 0.608). Partial ablation short-term success (OR 0.097; 95% CI 0.010–0.980; *p* = 0.048) and complete ablation short-term success (OR 0.140; 95% CI 0.024–0.822; *p* = 0.029) were predictors of non-inducibility during NIPS in the whole cohort. However, VT inducibility in NIPS before discharge was not associated with VT recurrence (HR 1.443; 95% CI 0.681–3.058; *p* = 0.338), irrespective of ES (HR 0.843; 95% CI 0.280–2.539; *p* = 0.762) or non-ES patients (HR 1.236; 95% CI 0.399–3.834; *p* = 0.714). Additionally, rehospitalization rates (HR 0.979; 95% CI 0.535–1.794; *p* = 0.946) during follow-up were not associated with VT inducibility during NIPS in ES (HR 0.728; 95% CI 0.305–1.736; *p* = 0.474) and non-ES patients (HR 0.832; 95% CI 0.338–2.046; *p* = 0.689).

## 4. Discussion

Data comparing short- and long-term outcomes of VT ablation in patients with ES compared to those with VT but without ES are scarce. In contrast to many other published studies, in our cohort, discontinuation of antiarrhythmic drugs was pursued in most patients, with 40% of ES and 20% of non-ES patients left on antiarrhythmic drugs at discharge. Together with our follow-up and keeping in mind that most VT recurrences occur relatively soon after VT ablation, our study delivers a realistic picture of the long-term ablation results in ES patients.

Catheter ablation of ES is safe in patients with structural heart disease and reveals comparable complication rates to VT ablation in non-ES patients. Nonetheless, ES patients were sicker, with worse LVEF values, higher rates of AAD therapy and higher rates of DCM as underlying heart disease. Accordingly, ES patients had higher rates of inducible VTs, hemodynamically not tolerated VTs and epicardial substrates, and were more frequently catecholamine-dependent during VT ablation. ES was identified as a negative predictor of acute ablation success. ES patients experienced higher VT recurrences, rehospitalizations and subsequent cardiovascular mortality rates, even after propensity score matching. Independent predictors of VT recurrence were the presence of ES, DCM and incomplete ablation success, whereas ES, pre-procedural imaging and decreased LVEF were predictors of rehospitalization. Testing of the inducibility of VTs with NIPS before hospital discharge strongly correlated with the results at the end of the VT ablation procedure, but was not found to be predictive of VT recurrences during follow-up, irrespective of ES or non-ES patients.

### 4.1. Electrical Storm and Mortality

ES occurs frequently and unpredictably in patients with structural heart disease. However, most large studies investigating VT ablation included a relatively small portion of ES patients or were of small sample size. ES has been recognized as an important predictor of VT recurrences and subsequent cardiovascular death independently of other established prognostic factors such as LVEF [4,13,14,15]. It is a life-threatening condition affecting around 10–30% of all ICD patients and more than triples the risk for subsequent death [3,13,16]. The high mortality rates may be explained by the compound effects of the arrhythmia itself, the multiple ICD shocks in a short timeframe and the underlying advanced heart failure. The respective contributions of these factors are still under debate [16]. More than half of all sudden cardiac deaths in ICD recipients are tachyarrhythmia-related [17]. Multiple shocks might contribute to transient systolic dysfunction and acute heart failure in terms of cardiac decompensation. Sweeney et al. showed increased mortality rates among patients with VT/VF terminated by shocks compared to patients treated only with anti-tachycardia pacing or without antiarrhythmic therapy [18]. Bänsch et al. propose recurrent VTs as a major driver for the increase in mortality, as they cause and promote LV dysfunction leading to end-stage heart failure, cardiogenic shock and death [13]. Accordingly, the results of the present study deliver further insights and enforce the hypothesis that ES might affect mortality via LV dysfunction since ES patients presented with significantly decreased LVEF. The prevention of recurrent VT episodes with successful VT ablation might attenuate the detrimental effect of arrhythmic episodes on mortality, with significantly better survival of ES patients without recurrent VT episodes compared to those with ongoing VTs [15].

### 4.2. Electrical Storm and VT Ablation

Several studies reported outcome data after catheter ablation in ES patients [19,20]. The landmark paper of Carbucicchio et al. included 95 patients with structural heart disease and ES and could show that after one to three procedures, a suppression of the clinical VT was achieved in 89% of patients. After 22 months, 92% of all patients were free from ES and 66% free from VT recurrences [19]. However, this study, just as most others, did not make a comparison to non-storm patients, and outcome specifics in relation to the underlying structural heart disease are lacking. In our study, the long-term ablation success was lower, with freedom from ES recurrences in 86% and freedom from any VT recurrences in only 35% of cases, despite comparable acute ablation success at the end of the procedure (76% vs. 72%). Notably, our study included more than half of ES patients with NICM (compared to 25% in Carbucicchio et al.), and long-term success tends to be lower in NICM patients [21]. On the other hand, we only found a statistical trend for ES patients with NICM for increased VT recurrences, with comparable outcomes in regard to rehospitalization rates and mortality rates. Possible reasons might be late presentation, more advanced heart failure or more difficult substrate targets. The combination of all these factors is likely to explain the worse prognosis of ES patients [21]. Recurrent ES episodes after the initial storm and after catheter ablation are associated with even worse outcomes after follow-up [20,22]. These findings could not be confirmed in our analysis. Notably, in univariate regression analysis, discharge with amiodarone was associated with increased VT recurrences. Patients with amiodarone had the clinically highest pretest probability of recurrent VTs and were, therefore, discharged with amiodarone.

### 4.3. Value of Programmed Electrical Stimulation after VT Ablation in ES Patients

Long-term outcomes of catheter ablation remain suboptimal, with recurrence rates over 50% during follow-up in randomized controlled trials. Currently, programmed stimulation at the end of the ablation procedure is recommended as the gold standard, with non-inducibility as the only endpoint [23]. Patients still with inducible VTs are considered at high risk of recurrence, which is in line with our results in which incomplete ablation success was an independent predictor of VT recurrences during follow-up. However, programmed stimulation at the end of the procedure has several limitations including haemodynamically instability, alterations in autonomic tone or regressions of ablation lesions. Therefore, non-inducibility beyond the acute phase before discharge was proposed as additional endpoint of VT ablation. This is the first study investigating the prognostic value of NIPS in consecutive ES patients. VT inducibility before discharge in ES patients did not correlate with NIPS at the end of the procedure and before discharge nor with prognostic endpoints, irrespective of ICM or NICM patients. Several studies report conflicting results for non-ES patients after VT ablation, partly investigating the impact of complete VT ablation, partly the impact of ablation of the clinical VT only [24,25,26]. Muser et al. found persistent VT inducibility during NIPS in NICM patients to be an independent predictor of recurrences [26]. NIPS at a median of 6 days after VT ablation was found to be a more accurate predictor of recurrences than programmed electrical stimulation at the end of the procedure (positive and negative predictive values 53% and 88% vs. 43% and 71%, respectively), which might explain the conflicting results of our study with a median of 3 days between VT ablation and NIPS [27]. It can be speculated that prolongation of the period between ablation and NIPS increases the prognostic potential of NIPS.

## 5. Study Limitations

This study is a single-centre, non-randomized study representing the experience of a German high-volume EP centre. We serve as a tertiary referral centre for VT ablations and, as such, it is possible that there is a referral bias that may limit the generalizability of our results. This study represents the outcomes after a single VT ablation procedure, and repeated VT ablation could change long-term outcomes. This study represents a patient cohort with advanced heart failure and severely reduced left ventricular function. Patients with an earlier disease state may be different from those included in this study. NIPS before discharge was not performed in all patients and generalizability of the present results could, therefore, be limited, especially because hemodynamically very unstable patients were usually not tested with NIPS. If the clinical VT is still inducible at the end of the ablation procedure or patients present spontaneous VT recurrence during hospitalization, NIPS is unnecessary. Myocardial biomarkers were taken upon hospital admission, but not all patients were transferred to the hospital directly after VT/ES episodes and their clinical significance is, therefore, unclear.

## 6. Conclusions

Catheter ablation of ES is safe and effective for the suppression of recurrent ES episodes; however, it reveals worse short-term as well as worse long-term success rates compared no non-ES VT ablations. ES patients with DCM and only partially successful ablation procedure represent a population with the highest risk for VT recurrences. The aiming of complete non-inducibility of all VTs should be adopted in ES patients.

## Figures and Tables

**Table 1:** Baseline characteristics.

	Before Matching (n = 311)					After Matching (n = 184)				
Characteristic	ES (n = 108; 35%)		Non-ES (n = 203; 65%)		*p* Value	ES (n = 92; 50%)		Non-ES (n = 92; 50%)		*p* Value
Age, median (range)	65 ± 14		62 ± 15		0.165	65 ± 13		62 ± 14		0.279
Males, *n* (%)	93	(86)	174	(86)	0.924	79	(86)	80	(87)	0.830
Cardiovascular risk factors, *n* (%)										
Arterial hypertension	85	(79)	159	(78)	0.820	76	(84)	72	(78)	0.366
Diabetes mellitus	33	(35)	46	(23)	0.056	33	(36)	25	(27)	0.186
Hyperlipidemia	72	(67)	135	(67)	0.889	62	(68)	60	(65)	0.676
Smoking	32	(30)	72	(36)	0.324	28	(31)	34	(37)	0.377
Cardiac family history	16	(15)	38	(19)	0.865	14	(15)	13	(14)	0.811
Comorbidities, *n* (%)										
Atrial fibrillation	47	(44)	77	(38)	0.338	37	(40)	36	(39)	0.880
Stroke	11	(10)	20	(10)	0.905	10	(11)	9	(10)	0.789
Chronic kidney disease	54	(51)	88	(43)	0.232	44	(48)	43	(47)	0.827
Liver cirrhosis	0	(0)	7	(3)	**0.050**	0	(0)	4	(4)	**0.044**
COPD	3	(3)	16	(8)	0.061	3	(3)	7	(8)	0.206
Asthma	0	(0)	3	(2)	0.279	0	(0)	1	(1)	0.319
Structural heart disease, *n* (%)										
Ischemic cardiomyopathy	49	(45)	108	(53)	0.188	46	(50)	42	(46)	0.555
Dilated cardiomyopathy	33	(31)	35	(17)	**0.007**	24	(26)	23	(25)	0.866
Myocarditis	13	(12)	26	(13)	0.845	11	(12)	13	(14)	0.622
Sarcoidosis	6	(6)	9	(4)	0.660	5	(5)	8	(9)	0.388
ARVC	2	(2)	8	(4)	0.503	1	(1)	3	(3)	0.312
HCM	2	(2)	2	(2)	0.432	2	(2)	1	(1)	0.560
HOCM	2	(2)	2	(2)	0.432	1	(1)	0	(0)	1.000
EMAH	0	(0)	3	(2)	0.277	-	-	-	-	-
Idiopathic	2	(2)	12	(6)	0.082	1	(1)	3	(3)	0.312
Cardiac biomarker										
Potassium (mmol/L)	4.33 ± 0.56		4.48 ± 0.51		**0.019**	4.30 ± 0.57		4.50 ± 0.49		**0.014**
Troponine (ng/mL)	0.211 ± 0.546		0.190 ± 0.435		0.845	0.240 ± 0.593		0.080 ± 0.173		0.068
proBNP (pg/mL)	2174 ± 1910		2048 ± 2150		0.707	2201 ± 1954		1947 ± 1727		0.518
Medication at admission, *n* (%)										
Beta blocker	94	(89)	181	(90)	0.803	78	(87)	82	(89)	0.610
Amiodarone	57	(54)	46	(23)	**0.001**	45	(50)	27	(29)	**0.004**
Other AAD	6	(6)	2	(1)	**0.021**	3	(3)	1	(1)	0.296
LVEF, %	33 ± 14		39 ± 15		**0.001**	34 ± 14		36 ± 14		0.440
Type of ICD, *n* (%)										
ICD	60	(57)	102	(50)	0.372	49	(53)	45	(49)	0.555
CRT-D	27	(25)	24	(12)	**0.003**	23	(25)	15	(16)	0.145
s-ICD	1	(1)	4	(2)	0.432	1	(1)	2	(2)	0.560
ICD indication, *n* (%)										
Primary prevention	58	(62)	105	(66)	0.640	48	(61)	51	(68)	0.349
Secondary prevention	35	(38)	54	(34)		31	(39)	24	(32)	

AAD, antiarrhythmic drug; ACHD, adults with congenital heart defect; ARVC, arrhythmic right ventricular disease; COPD, chronic obstructive pulmonary disease; CRT-D, cardiac resynchronization therapy-defibrillator; ES, electrical storm; HCM, hypertrophic cardiomyopathy; HOCM, hypertrophic obstructive cardiomyopathy; ICD, implantable cardioverter defibrillator; LVEF, left ventricular ejection fraction. Bold values indicate statistical significance.

**Table 2:** Procedural data and intraprocedural success.

	Before Matching (*n* = 311)					After Matching (*n* = 184)				
Characteristic	ES (*n* = 108; 35%)		Non-ES (*n* = 203; 65%)		*p* Value	ES (*n* = 92; 50%)		Non-ES (*n* = 92; 50%)		*p* Value
Preprocedural imaging, *n* (%)	39	(36)	103	(51)	0.137	33	(36)	43	(47)	0.134
MRI, *n* (%)	18	(17)	34	(17)	0.985	16	(17)	30	(33)	**0.017**
CT, *n* (%)	21	(19)	69	(34)	**0.007**	17	(19)	13	(14)	0.425
Epicardial ablation, *n* (%)	29	(27)	26	(13)	**0.001**	19	(21)	21	(23)	0.721
Noninducible with PES, *n* (%)	17	(16)	56	(28)	**0.019**	16	(17)	19	(21)	0.573
VTs inducible, *n*/patient	1.9 ± 1.6		1.5 ± 1.5		**0.033**	1.8 ± 1.6		1.7 ± 1.6		0.927
Clinical VT CL, ms	368 ± 86		350 ± 85		0.136	364 ± 84		357 ± 85		0.653
Procedural duration, min	147 ± 44		138 ± 49		0.094	143 ± 40		150 ± 53		0.354
Fluoroscopy duration, min	15.3 ± 10.0		12.3 ± 8.3		**0.018**	13.5 ± 7.6		13.4 ± 7.9		0.963
Ablation time, min	31.0 ± 17.5		28.8 ± 43.1		0.531	29.9 ± 16.9		35.8 ± 60.9		0.387
Partial ablation success, *n* (%)	102	(94)	194	(95)	0.663	86	(94)	85	(92)	0.774
Hemodynamic not tolerated VT, *n* (%)	37	(35)	43	(21)	**0.010**	28	(30)	25	(27)	0.625
Catecholamine, *n* (%)	17	(16)	10	(5)	**0.001**	14	(15)	7	(8)	0.105
Intubation, *n* (%)	8	(8)	2	(1)	**0.004**	7	(8)	2	(2)	0.087
Full ablation success, *n* (%)	79	(76)	177	(91)	**0.001**	77	(84)	75	(82)	0.697
Beta blocker at discharge, *n* (%)	100	(95)	191	(95)	0.798	84	(94)	85	(93)	0.785
Amiodaron at discharge, *n* (%)	43	(41)	38	(19)	**0.001**	32	(36)	26	(29)	0.289

CL, cycle length; CT, computer tomography; ES, electrical storm; MRI, magnetic resonance imaging; PES, programmed electrical stimulation; VT ventricular tachycardia. Bold values indicate statistical significance.

**Table 3:** Complications.

Characteristic	ES (*n* = 108; 35%)		Non-ES (*n* = 203; 65%)		*p* Value
Major complications, *n* (%)	12	(11)	15	(7)	0.267
Vascular access-related	1	(1)	2	(1)	1.000
Third-degree AV block	4	(2)	5	(5)	0.504
Pneumonia	1	(1)	3	(2)	1.000
Cardiogenic shock	3	(3)	2	(1)	0.346
Pneumothorax	2	(2)	1	(1)	0.277
Stroke	1	(1)	1	(1)	1.000
In-hospital mortality, *n* (%)	2	(1)	1	(1)	0.277

AV, atrio-ventricular; ES, electrical storm.

**Table 4:** Primary and secondary endpoints.

	Before Matching (*n* = 311)					After Matching (*n* = 184)				
Characteristic	ES (*n* = 108; 35%)		Non-ES (*n* = 203; 65%)		*p* Value	ES (*n* = 92; 50%)		Non-ES (*n* = 92; 50%)		*p* Value
Primary endpoint, *n* (%)										
VT recurrence	54	(65)	71	(40)	**0.001**	48	(66)	35	(42)	**0.003**
Secondary endpoints, *n* (%)										
First rehospitalization, overall	66	(73)	90	(48)	**0.001**	58	(76)	46	(54)	**0.003**
VT	49	(54)	62	(32)	**0.001**	44	(54)	32	(38)	**0.037**
Acute heart failure	12	(13)	23	(12)	0.954	10	(13)	11	(12)	0.885
Acute myocardial infarction	3	(3)	0	(0)	**0.041**	3	(4)	0	(0)	0.116
Stroke	0	(0)	3	(2)	0.554	0	(0)	2	(2)	0.497
LVAD/HTX	2	(2)	1	(1)	0.277	1	(1)	1	(1)	0.979
Cardiovascular mortality	17	(18)	18	(9)	**0.040**	13	(16)	8	(9)	0.178

ES, electrical storm; HTX, heart transplantation; LVAD, left ventricular assist device; MACE, major adverse cardiac event; VT, ventricular tachycardia. Bold values indicate statistical significance.

**Table 5:** (A) Multivariable Cox regression for VT recurrences. (B) Uni- and multivariable Cox regression for rehospitalization. (C) Multivariable Cox regression for long-term cardiovascular mortality.

**(A)**	**Univariable**			**Multivariable**		
**Variable**	**HR**	**95% CI**	***p*** **Value**	**HR**	**95% CI**	***p*** **Value**
Age	1.002	0.990–1.015	0.716	-	-	-
Epicardial ablation	1.343	0.866–2.083	0.188	-	-	-
Any VT inducible	1.912	1.235–2.961	**0.004**	1.590	1.016–2.488	**0.042**
Clinical VT inducible	1.266	0.590–2.719	0.545	-	-	-
LVEF	1.006	1.004–1.132	0.298	-	-	-
DCM	1.953	1.324–2.883	**0.001**	1.808	1.207–2.709	**0.004**
Beta blocker at discharge	0.938	0.437–2.011	0.869	-	-	-
Amiodarone at discharge	1.728	1.187–2.515	**0.004**	-	-	-
Preprocedural imaging	0.738	0.518–1.051	0.092	-	-	-
ES	1.841	1.289–2.628	**0.001**	1.621	1.112–2.362	**0.012**
**(B)**	**Univariable**			**Multivariable**		
**Variable**	**HR**	**95% CI**	***p*** **Value**	**HR**	**95% CI**	***p*** **Value**
Age	1.005	0.993–1.016	0.431	-	-	-
Epicardial ablation	1.402	0.941–2.090	0.097	-	-	-
Any VT inducible	1.487	0.984–2.246	0.059	-	-	-
Clinical VT inducible	1.338	0.724–2.473	0.353	-	-	-
LVEF	1.038	1.010–1.075	**0.001**	1.032	1.010–1.045	**0.009**
DCM	1.952	1.370–2.780	**0.001**	-	-	-
Beta blocker at discharge	1.108	0.554–2.257	0.778	-	-	-
Amiodarone at discharge	1.831	1.310–2.560	**0.001**	-	-	-
Preprocedural imaging	0.734	0.534–1.009	0.057	0.630	0.453–0.876	**0.006**
ES	1.948	1.415–2.682	**0.001**	1.582	1.127–2.219	**0.008**
**(C)**	**Univariable**			**Multivariable**		
**Variable**	**HR**	**95% CI**	***p*** **Value**	**HR**	**95% CI**	***p*** **Value**
Age	1.090	1.050–1.131	**0.001**	1.101	1.054–1.149	**0.001**
Epicardial ablation	0.793	0.307–2.043	0.630	-	-	-
Any VT inducible	1.636	0.710–3.771	0.248	-	-	-
Clinical VT inducible	2.208	0.773–6.308	0.139	-	-	-
LVEF	1.145	1.030–1.325	**0.001**	1.102	1.023–1.278	**0.004**
DCM	1.240	0.581–2.646	0.579	-	-	-
Beta blocker at discharge	1.738	0.237–12.752	0.587	-	-	-
Amiodarone at discharge	2.692	1.344–5.395	**0.005**	-	-	-
Preprocedural imaging	0.316	0.144–0.695	**0.004**	0.361	0.155–0.844	**0.019**
ES	1.948	1.004–3.780	**0.049**	-	-	-

CI, confidence interval; DCM, dilative cardiomyopathy; ES, electrical storm; HR, hazard ratio; LVEF, left ventricular ejection fraction; VT, ventricular tachycardia.

## Data Availability

Data is available from the corresponding author upon reasonable request.

## References

[1] Pedersen C.T., Kay G.N., Kalman J., Borggrefe M., Della-Bella P., Dickfeld T., Dorian P., Huikuri H., Kim Y.H., Knight B. (2014). EHRA/HRS/APHRS expert consensus on ventricular arrhythmias. Heart Rhythm.

[2] Villacastin J., Almendral J., Arenal A., Albertos J., Ormaetxe J., Peinado R., Bueno H., Merino J.L., Pastor A., Medina O. (1996). Incidence and clinical significance of multiple consecutive, appropriate, high-energy discharges in patients with implanted cardioverter-defibrillators. Circulation.

[3] Credner S.C., Klingenheben T., Mauss O., Sticherling C., Hohnloser S.H. (1998). Electrical storm in patients with transvenous implantable cardioverter-defibrillators: Incidence, management and prognostic implications. J. Am. Coll. Cardiol..

[4] Verma A., Kilicaslan F., Marrouche N.F., Minor S., Khan M., Wazni O., Burkhardt J.D., Belden W.A., Cummings J.E., Abdul-Karim A. (2004). Prevalence, predictors, and mortality significance of the causative arrhythmia in patients with electrical storm. J. Cardiovasc. Electrophysiol..

[5] Israel C.W., Barold S.S. (2007). Electrical storm in patients with an implanted defibrillator: A matter of definition. Ann. Noninvasive Electrocardiol..

[6] Israel C.W., Manegold J.C. (2014). Electrical storm: Definition, prevalence, causes and prognostic implications. Herzschrittmachertherapie + Elektrophysiologie.

[7] Priori S.G., Blomstrom-Lundqvist C., Mazzanti A., Blom N., Borggrefe M., Camm J., Elliott P.M., Fitzsimons D., Hatala R., Hindricks G. (2015). 2015 ESC Guidelines for the management of patients with ventricular arrhythmias and the prevention of sudden cardiac death: The Task Force for the Management of Patients with Ventricular Arrhythmias and the Prevention of Sudden Cardiac Death of the European Society of Cardiology (ESC). Endorsed by: Association for European Paediatric and Congenital Cardiology (AEPC). Eur. Heart J..

[8] Muser D., Liang J.J., Pathak R.K., Magnani S., Castro S.A., Hayashi T., Garcia F.C., Supple G.E., Riley M.P., Lin D. (2017). Long-Term Outcomes of Catheter Ablation of Electrical Storm in Nonischemic Dilated Cardiomyopathy Compared With Ischemic Cardiomyopathy. JACC Clin. Electrophysiol..

[9] Ponikowski P., Voors A.A., Anker S.D., Bueno H., Cleland J.G., Coats A.J., Falk V., Gonzalez-Juanatey J.R., Harjola V.P., Jankowska E.A. (2016). 2016 ESC Guidelines for the diagnosis and treatment of acute and chronic heart failure: The Task Force for the diagnosis and treatment of acute and chronic heart failure of the European Society of Cardiology (ESC). Developed with the special contribution of the Heart Failure Association (HFA) of the ESC. Eur. J. Heart Fail..

[10] Marchlinski F.E., Callans D.J., Gottlieb C.D., Zado E. (2000). Linear ablation lesions for control of unmappable ventricular tachycardia in patients with ischemic and nonischemic cardiomyopathy. Circulation.

[11] Jaïs P., Maury P., Khairy P., Sacher F., Nault I., Komatsu Y., Hocini M., Forclaz A., Jadidi A.S., Weerasooryia R. (2012). Elimination of local abnormal ventricular activities: A new end point for substrate modification in patients with scar-related ventricular tachycardia. Circulation.

[12] Sosa E., Scanavacca M., d’Avila A., Pilleggi F. (1996). A new technique to perform epicardial mapping in the electrophysiology laboratory. J. Cardiovasc. Electrophysiol..

[13] Bansch D., Bocker D., Brunn J., Weber M., Breithardt G., Block M. (2000). Clusters of ventricular tachycardias signify impaired survival in patients with idiopathic dilated cardiomyopathy and implantable cardioverter defibrillators. J. Am. Coll. Cardiol..

[14] Behnes M., Müller J., Ellguth D., Schupp T., Taton G., Reiser L., Engelke N., Reichelt T., Bollow A., Kim S.H. (2019). Electrical storm is associated with impaired prognosis compared to ventricular tachyarrhythmias. Int. J. Cardiol..

[15] Vergara P., Tung R., Vaseghi M., Brombin C., Frankel D.S., Di Biase L., Nagashima K., Tedrow U., Tzou W.S., Sauer W.H. (2018). Successful ventricular tachycardia ablation in patients with electrical storm reduces recurrences and improves survival. Heart Rhythm.

[16] Guerra F., Shkoza M., Scappini L., Flori M., Capucci A. (2014). Role of electrical storm as a mortality and morbidity risk factor and its clinical predictors: A meta-analysis. Europace.

[17] Pires L.A., Lehmann M.H., Steinman R.T., Baga J.J., Schuger C.D. (1999). Sudden death in implantable cardioverter-defibrillator recipients: Clinical context, arrhythmic events and device responses. J. Am. Coll. Cardiol..

[18] Sweeney M.O., Sherfesee L., DeGroot P.J., Wathen M.S., Wilkoff B.L. (2010). Differences in effects of electrical therapy type for ventricular arrhythmias on mortality in implantable cardioverter-defibrillator patients. Heart Rhythm.

[19] Carbucicchio C., Santamaria M., Trevisi N., Maccabelli G., Giraldi F., Fassini G., Riva S., Moltrasio M., Cireddu M., Veglia F. (2008). Catheter ablation for the treatment of electrical storm in patients with implantable cardioverter-defibrillators: Short- and long-term outcomes in a prospective single-center study. Circulation.

[20] Kozeluhova M., Peichl P., Cihak R., Wichterle D., Vancura V., Bytesnik J., Kautzner J. (2011). Catheter ablation of electrical storm in patients with structural heart disease. Europace.

[21] Dinov B., Fiedler L., Schönbauer R., Bollmann A., Rolf S., Piorkowski C., Hindricks G., Arya A. (2014). Outcomes in catheter ablation of ventricular tachycardia in dilated nonischemic cardiomyopathy compared with ischemic cardiomyopathy: Results from the Prospective Heart Centre of Leipzig VT (HELP-VT) Study. Circulation.

[22] Kumar S., Fujii A., Kapur S., Romero J., Mehta N.K., Tanigawa S., Epstein L.M., Koplan B.A., Michaud G.F., John R.M. (2017). Beyond the Storm: Comparison of Clinical Factors, Arrhythmogenic Substrate, and Catheter Ablation Outcomes in Structural Heart Disease Patients With versus Those Without a History of Ventricular Tachycardia Storm. J. Cardiovasc. Electrophysiol..

[23] Al-Khatib S.M., Stevenson W.G., Ackerman M.J., Bryant W.J., Callans D.J., Curtis A.B., Deal B.J., Dickfeld T., Field M.E., Fonarow G.C. (2018). 2017 AHA/ACC/HRS Guideline for Management of Patients With Ventricular Arrhythmias and the Prevention of Sudden Cardiac Death: Executive Summary: A Report of the American College of Cardiology/American Heart Association Task Force on Clinical Practice Guidelines and the Heart Rhythm Society. Circulation.

[24] Piers S.R., Leong D.P., van Huls van Taxis C.F., Tayyebi M., Trines S.A., Pijnappels D.A., Delgado V., Schalij M.J., Zeppenfeld K. (2013). Outcome of ventricular tachycardia ablation in patients with nonischemic cardiomyopathy: The impact of noninducibility. Circ. Arrhythmia Electrophysiol..

[25] Dinov B., Arya A., Schratter A., Schirripa V., Fiedler L., Sommer P., Bollmann A., Rolf S., Piorkowski C., Hindricks G. (2015). Catheter ablation of ventricular tachycardia and mortality in patients with nonischemic dilated cardiomyopathy: Can noninducibility after ablation be a predictor for reduced mortality?. Circ. Arrhythmia Electrophysiol..

[26] Muser D., Hayashi T., Castro S.A., Supple G.E., Schaller R.D., Santangeli P., Arkles J., Kumareswaran R., Nazarian S., Deo R. (2019). Noninvasive Programmed Ventricular Stimulation-Guided Management Following Ventricular Tachycardia Ablation. JACC Clin. Electrophysiol..

[27] Oloriz T., Baratto F., Trevisi N., Barbaro M., Bisceglia C., D’Angelo G., Yamase M., Paglino G., Radinovic A., Della Bella P. (2018). Defining the Outcome of Ventricular Tachycardia Ablation: Timing and Value of Programmed Ventricular Stimulation. Circ. Arrhythmia Electrophysiol..

